# Toxin-antitoxin genes are differentially expressed in *Escherichia coli relA* and *spoT* mutans cultured under nitrogen, fatty acid, or carbon starvation conditions

**DOI:** 10.3389/fmicb.2024.1528825

**Published:** 2025-01-17

**Authors:** Monika Maciąg-Dorszyńska, Paweł Olszewski, Monika Karczewska, Lidia Boss

**Affiliations:** ^1^Department of Bacterial Molecular Genetics, Faculty of Biology, University of Gdańsk, Gdańsk, Poland; ^2^Department of Neuroscience, Uppsala University, Uppsala, Sweden

**Keywords:** toxin-antitoxin, *relA*, *spoT203*, ppGpp, RNA-seq, *hokB*, *mqsRA*, *ghoST*

## Abstract

**Introduction:**

The stringent response is one of the fundamental mechanisms that control and modulate bacterial adaptation to stress conditions, such as nutrient limitation. The accumulation of stringent response effectors, (p)ppGpp, causes differential expression of approximately 500 genes, including genes of bacterial endogenous toxin-antitoxin (TA) systems. However, the exact link between (p)ppGpp and toxin-antitoxin systems’ activation, as well as toxin-antitoxin role in stress adaptation remains disputed.

**Methods:**

In this study, we performed a complex analysis of changes (RNA-Seq) in the toxin-antitoxin operons’ transcription in response to nitrogen, fatty acid, or carbon starvation, in bacteria with different abilities of (p)ppGpp accumulation.

**Results and discussion:**

Although we observed that in some cases (p)ppGpp accumulation appears to be crucial for transcriptional activation of TA genes (e.g., *ghoST, ryeA*), our data indicates that the general pattern of chromosomally encoded TA gene expression in *E. coli* differs depending on the nutrient distribution in the environment, regardless of the alarmone accumulation.

## Introduction

The availability of nutrients is a key element regulating the cell’s entire metabolism; thus, the deficiency of nutrients activates specific adaptation mechanisms. The stringent response is one of the fundamental mechanisms that control and modulate adaptation to stress conditions in bacteria. The stringent response effectors are specific nucleotides, guanosine tetraphosphate and guanosine pentaphosphate, collectively abbreviated as (p)ppGpp. These nucleotides, in *E. coli*, are synthesized by RelA and SpoT proteins using two different pathways, where RelA produces (p)ppGpp in response to the presence of uncharged tRNA in the ribosomal A-site, during amino acid starvation, or in response to pyruvate depletion during fatty acid starvation (Kushwaha et al., 2019; Sinha et al., 2019). On the other hand, SpoT is responsible for accumulation of (p)ppGpp in response to glucose or fatty acid starvation and several other stress conditions (Potrykus and Cashel, 2008). Moreover, SpoT also acts as a (p)ppGpp hydrolase (Potrykus and Cashel, 2008).

The (p)ppGpp nucleotides, acting as cellular alarmones, regulate various crucial cellular processes such as: transcription, translation, and initiation of DNA replication, directly and indirectly. In *E. coli* (p)ppGpp exerts its effect mainly by binding to the core subunits of the RNA polymerase to modulate its transcriptional properties, affecting in this way numerous processes (Irving et al., 2021). Accumulation of (p)ppGpp causes differential expression of approximately 500 genes (Costanzo and Ades, 2006; Traxler et al., 2008, 2011), including toxin-antitoxin (TA) encoding genes (Christensen-Dalsgaard et al., 2010; Shan et al., 2017; LeRoux et al., 2020). Beside RelA/SpoT homologs (RSH), many bacteria encode additional enzymes engaged in alarmone biosynthesis and hydrolysis, namely small alarmone synthetases (SAS) and small alarmone hydrolases (SAH), which makes the model of (p)ppGpp-mediated regulation of transcription even more complicated (Potrykus and Cashel, 2018). Surprisingly, in 2020 it has even been proposed that some of the small alarmone synthetases (SAS) from *Cellulomonas marina* can itself act as a toxic part of TA systems (Jimmy et al., 2020).

Since the mid-80s TA systems were considered to be linked, along with the (p)ppGpp alarmone, to bacterial persistent phenotype (Pacios et al., 2020; Brown, 2019; Niu, 2024). Persister cells are a subpopulation of bacteria that are nongrowing, dormant bacteria exhibiting transient high levels of tolerance to antibiotics (Pacios et al., 2020). However, a few years ago the hypothesis about TA systems’ role in persister cell formation was challenged (Van Melderen and Wood, 2017; Pacios et al., 2020; Brown, 2019; Niu, 2024). Additionally, although numerous studies have reported TA operons’ transcriptional activation in response to diverse stress conditions (Christensen-Dalsgaard et al., 2010; Muthuramalingam et al., 2016; Shan et al., 2017; Ronneau and Helaine, 2019; LeRoux et al., 2020), this was not necessarily accompanied by detection of a given toxin activity (LeRoux et al., 2020). Therefore, the link between the stringent response and TA systems’ activity remains elusive (Fraikin et al., 2019; LeRoux et al., 2020; Sanchez-Torres and Wood, 2024).

To shed some light on the linkage between (p)ppGpp and transcriptional regulation of toxin-antitoxin systems, we performed a complex RNA-seq analysis of *E. coli* bearing *relA* and *spoT* mutations, cultured under different limited nutritional conditions. We noted expression of 28 TA operons from 35 experimentally validated and one bioinformatically predicted in *E. coli* K-12 MG1655 strain (Guan et al., 2024). We find that, among them the expression of 20 TA operons is not significantly changed (−2 > log2FD > 2), or statistically insignificant, whereas several TA genes are differentially expressed in response to carbon or fatty acid starvation in the tested strains. According to our data (p)ppGpp appears to affect expression of several TA systems (*ghoST*, *relBE*, *ryeA, yefM-yoeB, chpBS, topAi-yjhQ*), while the expression of other TA genes (*tisB*, *hokB*, *mqsR*) seems to be independent of the alarmone accumulation. Therefore, we propose that alterations in intracellular (p)ppGpp levels cannot be considered as the main element of transcriptional regulation of all TA systems.

## Materials and methods

### Bacterial growth and RNA-Seq

All experiments were conducted using the *Escherichia coli* K-12 MG1655 strain bearing a single *relA* deletion (*ΔrelA*), double deletion of the *relA* and *spoT* genes (*ΔrelA ΔspoT*), or *ΔrelA spoT203* mutations (Xiao et al., 1991; Jishage et al., 2002; Potrykus et al., 2011). Bacteria were grown to the early logarithmic phase in M9 medium supplemented with glucose and casamino acids as carbon sources. Upon reaching OD_600_ = 0.2, carbon, nitrogen, and fatty acid starvation were induced. To induce carbon and nitrogen stress, cells were centrifuged at 4,000 × g for 10 min and then resuspended in fresh medium lacking carbon sources (glucose and casamino acids). For nitrogen starvation, following centrifugation, bacteria were cultured in a nitrogen-limited minimal medium without (NH_4_)_2_SO_4_ as the nitrogen source. Fatty acids’ stress was induced by adding triclosan (2 μg/mL). RNA-seq samples were collected after 1.5 cell generations. A volume of 2.5 mL from each culture was collected and mixed with 5% AquaPhenol in 96% ethanol. The samples were then rapidly frozen in liquid nitrogen and stored at −80°C. For RNA extraction, the frozen samples were thawed on ice and centrifuged at 4,000 × g for 5 min. The cell pellet was resuspended in 100 μL of Tris-EDTA (TE) buffer (10 mM Tris-Cl, 1 mM EDTA, pH 8.0) containing lysozyme (1 mg/mL) and incubated at room temperature for 10 min. Subsequent purification was performed using the RNeasy Mini Kit (Qiagen GmbH, Hilden, Germany) following the manufacturer’s instructions. To ensure complete removal of DNA, a DNAse I (Qiagen) treatment was applied during the column-based purification process. RNA was eluted in 50 μL of RNase-free water, and the purified RNA samples were stored at −80°C until analysis. Quality was checked by employing Bioanalyzer 2100. The sequencing runs were conducted on the Illumina NovaSeq6000 platform. Thirty million pair-end reads per sample were assessed with 101 pb read length.

### RNA-Seq processing and analysis

Raw *fastq* files were processed with *fastp* software using default parameters (Chen et al., 2018). Reads were aligned to the *Escherichia coli* reference genome NC_000913.3 using *Rsubread* v2.6.4 package (Liao et al., 2019). In total, 99.5% of reads were uniquely mapped to the reference genome. Resulting BAM files were summarized into counts with *Rsubread* v2.6.4. Differential gene expression (DE) analysis was performed with *DESeq2* package v1.44.0 (Love et al., 2014). Adjusted *p* value for multiple testing was calculated with Benjamini-Hochberg correction. If not stated otherwise, significant DE genes were selected with |log2FC| ≥ 1 and adjusted *p* value < 0.05 thresholds. Analysis using median average deviation (MAD; Chung et al., 2008) used median ± 3*MAD. GO terms and KEGG pathways were analyzed with *clusterProfiler* package v 4.12.6 (Yu et al., 2012). Heat maps were generated with *pheatmap* package v1.0.12 and volcano plots were generated with *EnhancedVolcano* package v1.22.0. In all analyses R version 4.4.1 was used.

## Results and discussion

### No significant changes in TA transcription were observed during nitrogen starvation regardless of the (p)ppGpp intracellular level

**Figure 1:**
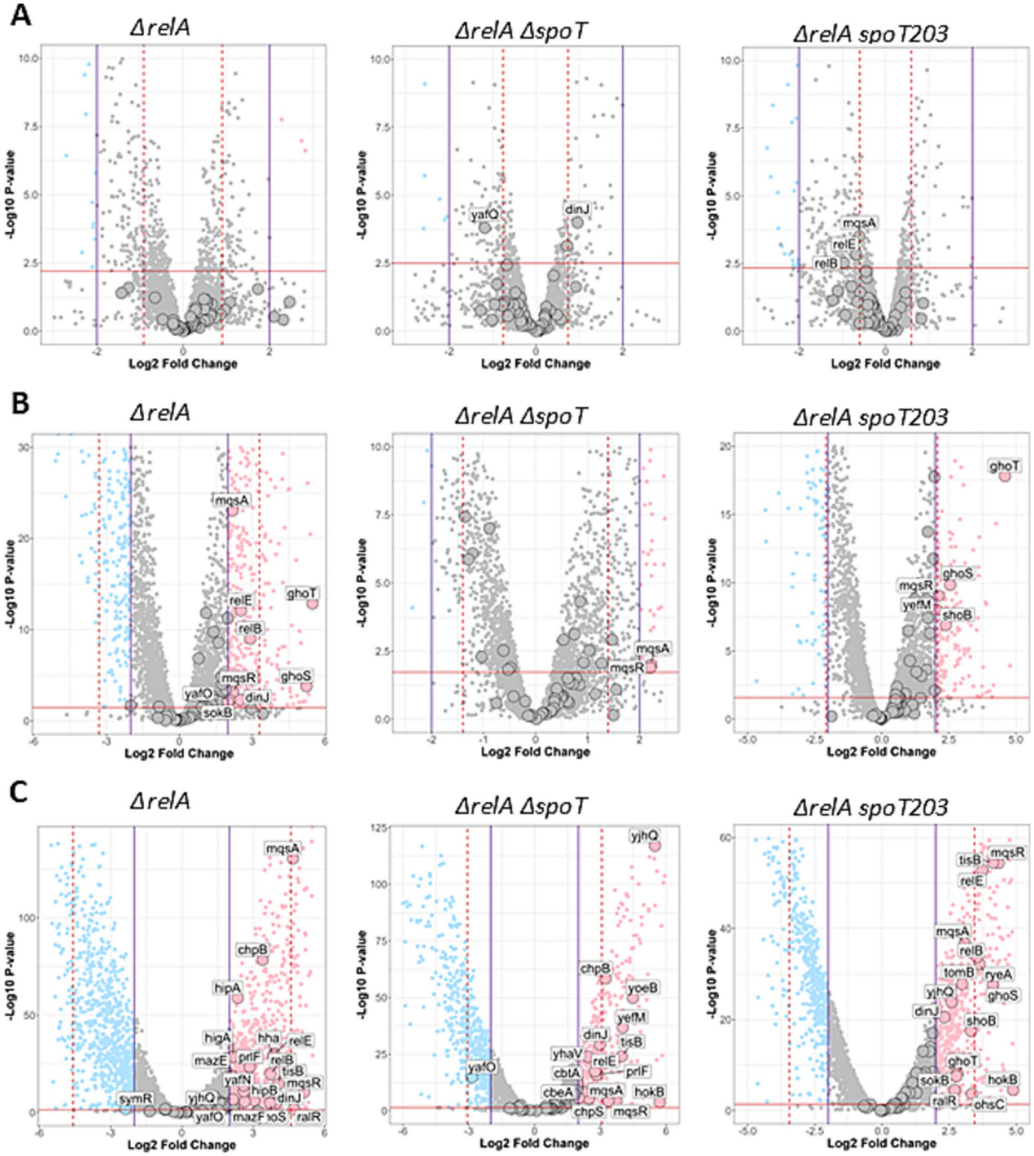
Overview of gene expression changes in *ΔrelA*, *ΔrelA ΔspoT*, and *ΔrelA spoT203* mutants upon nutritional stress. Stress conditions: nitrogen **(A)**, fatty acid deprivation **(B)**, and carbohydrate deprivation (NH^4+^) deprivation **(C)** compared to no stress (normal). Volcano plots illustrate log2 fold change (x-axis) and statistical significance of changes (y-axis) as calculated by DESeq2 package. Horizontal dashed red line indicates *p* value corresponding to adjusted p value = 0.05. Purple vertical lines indicate casual log2FC = 2 threshold and red dashed vertical lines indicate MAD thresholds calculated for each comparison. Genes which are significantly up or downregulated (|log2FC| >2 and adj. *p* < 0.05) are colored in pink and blue respectively, whereas genes colored in gray are not significant. TA genes are indicated by large points. Labels are shown only for significantly altered TA genes.

To test the effect of different intracellular (p)ppGpp level on TA operons’ transcriptional regulation, we assessed the whole transcriptome of three *E. coli* strains. Bacteria bearing double *ΔrelA ΔspoT* mutations (so called ppGpp^0^ or null strain) are unable to synthesize (p)ppGpp under any conditions; the *ΔrelA* mutant, which bears an active SpoT and a deletion of the *relA* gene, allows for the complete elimination of the effects caused by amino acid starvation; and *ΔrelA spoT203* is a strain with physiologically approximately sevenfold higher basal (p)ppGpp level when compared to the wild type strain (Sarubbi et al., 1988). Surprisingly, we find that there are no significant differences (−2 < log2FD < 2) in TA transcription in all tested strains cultured under nitrogen limiting conditions irrespectively of the bacterial ability of (p)ppGpp accumulation (Figure 1).

### The *mqsRA* and *ghoST* operons are differentially expressed in bacteria with varying SpoT activity during fatty acid starvation

**Figure 2:**
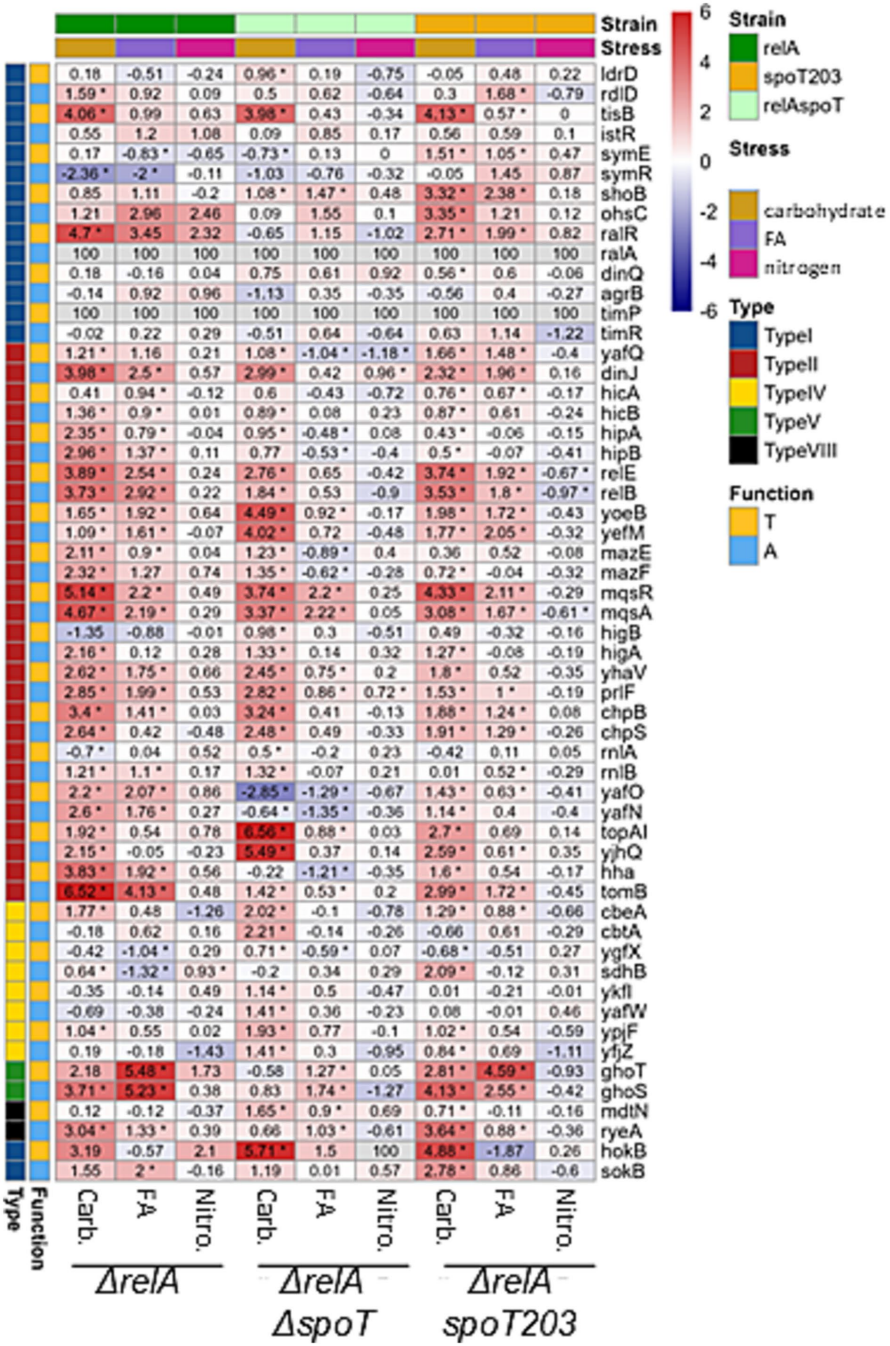
Changes in Toxin and Antitoxin Transcript Levels Due to Nutritional Stress. The heatmap illustrates the log2 fold change (log2FC) in transcript levels of toxin-antitoxin (TA) systems in cells subjected to various nutritional stresses for 1.5 generations, as assessed by Illumina NGS RNA-seq. Stress conditions: carbohydrate deprivation (Carb.), fatty acid deprivation (FA), and nitrogen (NH^4+^) deprivation (Nitro.) compared to no stress (normal). Mutant Strains: *ΔrelA*; *ΔrelA*, *ΔspoT* (ppGpp null); *ΔrelA*, *spoT203* (ppGpp high). Top Sidebar: Indicates the stress condition and mutant strain and left Sidebar: Indicates TA type and gene function (antitoxin or toxin). Color scale represents the exact log2FC values for the comparison of stress vs. normal conditions. Log2FC values for genes, mutants and conditions are provided in cells. Asterisk indicates statistical significance evaluated by DESeq2, adjusted *p* < 0.05. Small RNAs *ralA* and *timA* were not annotated in the genome version used in this study thus data for them were not available (n.a.).

Induction of fatty acid starvation significantly affected the transcription of several TA modules in tested bacteria, including *mqsRA*, *ghoST*, *hokB*-*sokB*, *relBE*, *dinJ-yafQ*, *tomB-hha* in bacteria bearing *ΔrelA* mutation, *mqsRA* in ppGpp^0^ strain, and *mqsRA*, *ghoST*, *yefM-yoeB* and *shoB* in *ΔrelA ΔspoT203* double mutant (Figures 1, 2). Among those, the expression pattern of two TA modules was particularly interesting.

Firstly, the expression of toxin-encoding gene *mqsR*, which was significantly upregulated (log2FC > 2) in all tested strains growing under fatty acid limiting conditions (Figures 1, 2), suggesting that its transcriptional regulation can be independent of (p)ppGpp accumulation. Since toxin and antitoxin transcription is usually coupled, often the upregulation of toxin is accompanied by increased antitoxin expression. However, in the strain with elevated intracellular alarmone level (*ΔrelA spoT203*), we noted significant *mqsR* gene upregulation (log2FC = 2.11) and only minor changes in *mqsA* transcription (log2FC = 1.67) (Figures 1, 2). This could suggest MqsR toxin activation via toxin mRNA enrichment and, as a result, unbalanced T:A intracellular ratio. Still, not without controversies, MqsA has been shown to possess a role in repression of the general stress response and curli/biofilm formation, while MqsR was shown to play a role in bile-acid stress reduction, biofilm formation, phage defense and regulation of type V *ghoST* TA system (Sanchez-Torres and Wood, 2024). In the proposed model, in the presence of MqsR, the *ghoS*-encoding 5′ region of the *ghoST* transcript is degraded, thereby enriching for the *ghoT*-encoding 3′ region (Wang et al., 2013). Interestingly, besides changes in the *mqsRA* genes expression, we also observed upregulation of *ghoST* system in the *ΔrelA* and *ΔrelA spoT203* strains. Curiously, we noted a distinctive pattern of *ghoST* expression in bacteria with (p)ppGpp excess (*ΔrelA spoT203*): the GhoT toxin transcription level was almost twice as high as GhoS antitoxin (log2FC 4.59 vs. 2.55) (Figures 1, 2). This indirectly confirms the MqsR-mediated mechanism of post-transcriptional regulation of GhoT activity under stress conditions.

To sum up, we noted upregulation of the whole *mqsRA* system in *ΔrelA* and ppGpp^0^ strains, and imbalance in MqsR toxin and MqsA antitoxin expression in *ΔrelA spoT203*, during fatty acid starvation. Elevated *mqsR* transcription was accompanied by unbalanced *ghoS* and *ghoT* transcription only in *ΔrelA spoT203* strain. Therefore, we suppose that although the upregulation of *mqsR* gene appears to be independent of (p)ppGpp accumulation, the activation of MqsR toxin and further regulation of *ghoST* expression require high intracellular (p)ppGpp level.

The *mqsRA* operon is negatively autoregulated by an antitoxin binding directly to its promoter region, and toxin and antitoxin encoding genes are co-transcribed (Brown et al., 2013). Thus, the unbalanced toxin and antitoxin mRNA level noted in this study appears to be enigmatic and requires further investigation, since there is a possibility of additional post-transcriptional regulation of *mqsRA*, e.g., by RNase-mediated degradation of *mqsA*-encoding mRNA during fatty acid starvation. In the recently proposed model, stress induces conformational changes in nascent and zinc-free MqsA, resulting in exposure of the ClpX recognition motif for ClpXP-mediated degradation of the antitoxin, leading to MqsR activation (Vos et al., 2022). We believe that the MqsR toxin activation may require both elevated (p)ppGpp level and other concomitant factors, such as MqsA degradation by protease and/or depletion of *mqsA* encoding mRNA.

### SpoT activity affects transcriptional regulation of some but not all TA systems under carbon starvation

After induction of carbon starvation, we observed significant changes (log2FC >3) in expression of several TA modules (Figures 1, 2), some of which were previously indicated as upregulated in response to stress conditions (*mqsRA*, *relBE*, *yefM-yoeB*, *hokB, tisB*) (Christensen et al., 2001; Wang et al., 2013; Janssen et al., 2015; Verstraeten et al., 2015; Su et al., 2022; Vos et al., 2022). In all tested strains, irrespective of SpoT activity, we noted *tisB*, *hokB* and *mqsRA* upregulation (Figures 1, 2), suggesting that the transcriptional activation of these genes is (p)ppGpp independent, at least under tested conditions.

One interesting observation was that the expression of *hokB* was negligible in all but carbohydrate stress conditions (Figures 1, 2). HokB is a toxin of a type I TA system, in which antitoxin is represented by a small antisense RNA (SokB) that binds the toxin mRNA and inhibits its translation. In the case of type I TA systems, transcriptional activation of only the toxin-encoding gene probably means that there are not enough antitoxin molecules to inhibit toxin translation, leading to its activation. Some data suggest that HokB activation may lead to persister cell formation (Yokoyama et al., 2023; Verstraeten et al., 2015). One of the proposed mechanisms of HokB-mediated persistence was based on the activity of the Obg protein that interacts with SpoT and stimulates (p)ppGpp synthetase activity of SpoT. According to this model, an elevated alarmone level leads to enhanced expression of the pore-forming HokB and, as a result, to a collapse of the membrane potential causing ATP leakage associated with persistence (Verstraeten et al., 2015). In fact, some data suggest that (p)ppGpp induction during carbon starvation affects bacterial persistence formation in the presence of ciprofloxacin (Svenningsen et al., 2021). However, we observed *hokB* upregulation in all tested bacterial strains in response to carbon starvation, including the ppGpp^0^ strain (log2FC = 5.71) (Figures 1, 2), which suggests that *hokB* transcriptional activation can be independent of (p)ppGpp intracellular level. Moreover, some studies claim that it is highly unlikely that this single gene would be the central element in the mechanism of persistent phenotype manifestation (Song and Wood, 2020). Especially since the *hok-sok loci* occasionally degenerate and appear to be non-functional in some bacterial strains (Fozo et al., 2010). Therefore, we suppose that the upregulation of *hokB* gene and possible toxin activation during growth in carbon limited environment is rather a secondary effect of more complexed bacterial response to nutritional limitation, than a conserved part of a specific pattern of bacterial response governed by (p)ppGpp under these environmental conditions.

One the other hand, recently novel biological functions were proposed for another TA system upregulated in our experiment. Namely, it appears that *tisB-istR* participates in blocking the import of host and/or microbial-produced genotoxic compounds, allowing for increased survival in the mammalian gut (Su et al., 2022). Moreover, it has been demonstrated that TisB toxin is involved in bacterial response to high doses of ofloxacin, even though it is not required for ofloxacin inhibitory effect (Cayron et al., 2024). Therefore, the subject of TA system influence on bacterial cell physiology remains elusive and should be addressed carefully.

Besides *tisB*, *hokB*, and *mqsRA*, only three TA systems were highly upregulated (log2FC > 3) after induction of carbon starvation in the ppGpp^0^ strain: *topAI-yjhQ*, *yefM-yoeB*, and *chpBS*. The toxin of the *topAI-yjhQ* system inhibits topoisomerase I activity (Yamaguchi and Inouye, 2015), while YoeB and ChpB toxins are endoribonucleases that probably affect ribosome biosynthesis (Culviner et al., 2020). However, it has been claimed that the upregulation of TA operons is not necessarily accompanied by the corresponding toxin activity (LeRoux et al., 2020), thus further studies are needed to make any definitive conclusions here. The *relBE* operon as well as *ghoS* and *ryeA* antitoxins were upregulated both in the *ΔrelA* and *ΔrelA spoT203* bacterial strains, but not in the ppGpp^0^ strain. Therefore, we conclude that transcriptional activation of *relBE*, *ghoST*, and *ryeA* under carbon starvation conditions requires (p)ppGpp synthesis. RyeA is a novel type VIII antitoxin (Choi et al., 2018) and has been shown to be most strongly expressed in minimal medium during exponential growth (Gupta et al., 2019) and upon acid exposure (Gupta et al., 2020). Recently, it has been demonstrated that during acid stress *ryeA-sdsR* system expression is controlled by a third factor—the GcvB small RNA (Ibrahim et al., 2024). It cannot be excluded that GcvB stabilizes the RyeA antitoxin also during carbon starvation and, since we detected no *ryeA* upregulation in (p)ppGpp deficient bacteria, that this protective effect is dependent on (p)ppGpp.

Since *relBE* system is encoded within DNA of the Qin cryptic prophage, its genetic localization suggests that this system’s activity could be connected to Qin DNA stabilization (Wang and Wood, 2016). Interestingly, we observed upregulation of some genes associated with the Qin prophage, including the *ydfD* gene (log2FC = 4.01 in *ΔrelA* and log2FC = 2.01 in ppGpp^0^ strain growing under fatty acids starvation, data not shown), encoding phage lysis protein (Lu et al., 2022). However, this observation seems to be devoid of correlation with the *relBE* expression, since no changes in *relBE* expression were observed in these conditions (Figures 1, 2).

**Figure 3:**
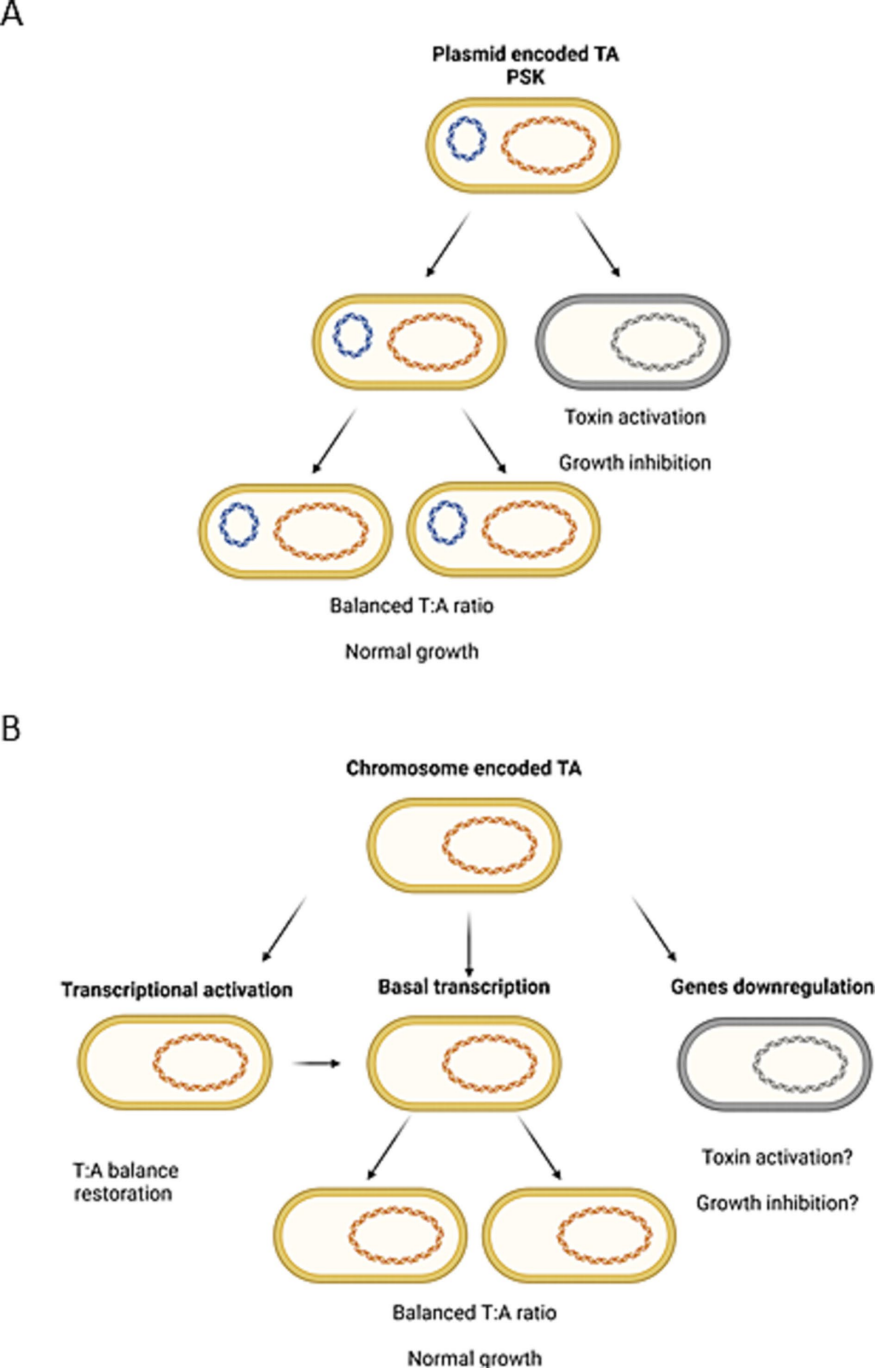
Illustration of the possible analogy between **(A)** the generally accepted mechanism of bacteria killing by plasmid-encoded toxin activation due to post-segregational killing (PSK) mechanism and **(B)** differential expression of chromosomally encoded TA systems. Created with Biorender.com.

To summarize, we observed that transcriptional activation of some TA genes during nutrient limitation seems to be independent of (p)ppGpp availability (*tisB*, *hokB*, *mqsR*), while the upregulation of others requires (p)ppGpp induction (e.g., *ghoST*, *relBE*, *ryeA*). In 2020 it has been claimed that although (p)ppGpp is sufficient to induce TA transcription, it was not essential for this induction (LeRoux et al., 2020). Moreover, it has been stated that the transcriptional activation of TA operons is not necessarily accompanied by a corresponding toxin activity (LeRoux et al., 2020). Our data agrees with these observations. We believe that nutritional stress or (p)ppGpp accumulation cannot be considered as universal factors inducing gene transcription of all TA systems resident within bacterial genome. Furthermore, we are convinced that transcriptional activation of a TA operon does not automatically lead to toxin activation. However, we suppose that in some cases (p)ppGpp induction can indirectly lead to alterations in the toxin and antitoxin ratio. The activity of a toxin can be the effect of imbalanced equilibrium of toxin and antitoxin molecules. This could be the result of an intensified toxin biosynthesis (e.g., due to enrichment in toxin mRNA), antitoxin preferential degradation by cellular proteases or both factors (reviewed in Boss and Kędzierska, 2023). In plasmid-encoded TA systems the post-segregational killing phenomenon (PSK) has been proven to exist (Fraikin and Van Melderen, 2024) as a result of uneven plasmid distribution into bacterial daughter cells (Figure 3A). In the case of chromosomal type II TA modules, it has been assumed frequently that since the TAs’ promoters are repressed by free antitoxin or TA complexes, if a toxin is activated, there should be less antitoxin and higher transcriptional activity of the whole operon (Christensen-Dalsgaard et al., 2010; Shan et al., 2017; LeRoux et al., 2020). In our opinion, that would be the moment when the toxin and antitoxin molecules distribution returns to the equilibrium state. Therefore, we think that to elucidate the mechanism of toxin activation, it is worth searching for conditions that trigger TA modules’ downregulation, since this would be the situation analogous to plasmid loss (Figure 3B). Nonetheless, we did not observe significant downregulation of any TA genes in tested bacteria during nutrient limitation (log2FC < −3, Figure 2). Therefore, this subject requires further research.

Overall, our data indicates that the general pattern of *E. coli* chromosomally encoded TA genes expression differs depending on the nutrients’ distribution in the environment, regardless of (p)ppGpp presence. The greatest number of TA genes is considerably changed (upregulated) in all tested strains after induction of carbon starvation, while the effect of fatty acid or nitrogen starvation is much less spectacular. It is worth keeping in mind that the regulation of gene expression covers both transcription and translation control. In 2018 it has been demonstrated that bacterial cells regulate these processes differently depending on which nutrient is being limited. For example, it has been shown that carbon starvation mostly affects the rate of transcription that is coupled to translation and thus slows down both processes. On the other hand, nitrogen starvation slows down translation rate independently of transcription, and translation seems to be uncoupled from transcription. Interestingly, it appears that during growth under nitrogen limited conditions, bacteria regulate translation rate by an unknown (p)ppGpp-independent mechanism (Iyer et al., 2018; Li et al., 2018; Potrykus and Cashel, 2018). Both situations described above seem to be reflected in our data. By contrast, generally TA systems are rather upregulated than downregulated when bacteria are cultured under nutrient limited conditions. However, it appears that modulation of TA gene expression in response to carbon starvation relates to modification of transcription level, while only a few TA modules were upregulated fatty acid limitation and no significant changes in TA transcription were observed after induction of nitrogen starvation. Furthermore, the role of (p)ppGpp in regulation of TA gene expression during nitrogen starvation was rather minor. Therefore, we hypothesize that under these conditions most of TA genes are either unaffected or regulated at the translation level, as proposed by Li et al. for other genes (Li et al., 2018).

## Data Availability

The original contributions presented in the study are included in the article/supplementary material, further inquiries can be directed to the corresponding author/s.
